# Early trauma-focused cognitive-behavioural therapy to prevent chronic post-traumatic stress disorder and related symptoms: A systematic review and meta-analysis

**DOI:** 10.1186/1471-244X-8-81

**Published:** 2008-09-19

**Authors:** Hege Kornør, Dagfinn Winje, Øivind Ekeberg, Lars Weisæth, Ingvild Kirkehei, Kjell Johansen, Asbjørn Steiro

**Affiliations:** 1 Norwegian Knowledge Centre for the Health Services, Box 7004 St. Olavplass, 0130 Oslo, Norway; 2 Department of Clinical Psychology, University of Bergen, Christiesgt 12, 5015 Bergen, Norway; 3 Department of Behavioural Sciences in Medicine, University of Oslo, Box 1111 Blindern, 0317 Oslo, Norway; 4 Norwegian Centre for Violence and Traumatic Stress Studies, Kirkev 166 Block 48, 0407 Oslo, Norway; 5 Haugev 3, 5005 Bergen, Norway

## Abstract

**Background:**

Early trauma-focused cognitive-behavioural therapy (TFCBT) holds promise as a preventive intervention for people at risk of developing chronic post-traumatic stress disorder (PTSD). The aim of this review was to provide an updated evaluation of the effectiveness of early TFCBT on the prevention of PTSD in high risk populations.

**Methods:**

We performed a systematic literature search in international electronic databases (MEDLINE, EMBASE, PsycINFO, CENTRAL, CINAHL, ISI and PILOTS) and included randomised controlled trials comparing TFCBT delivered within 3 months of trauma, to alternative interventions. All included studies were critically appraised using a standardised checklist. Two independent reviewers selected studies for inclusion and assessed study quality. Data extraction was performed by one reviewer and controlled by another. Where appropriate, we entered study results into meta-analyses.

**Results:**

Seven articles reporting the results of five RCTs were included. All compared TFCBT to supportive counselling (SC). The study population was patients with acute stress disorder (ASD) in four trials, and with a PTSD diagnosis disregarding the duration criterion in the fifth trial. The overall relative risk (RR) for a PTSD diagnosis was 0.56 (95% CI 0.42 to 0.76), 1.09 (95% CI 0.46 to 2.61) and 0.73 (95% CI 0.51 to 1.04) at 3–6 months, 9 months and 3–4 years post treatment, respectively. A subgroup analysis of the four ASD studies only resulted in RR = 0.36 (95% CI 0.17 to 0.78) for PTSD at 3–6 months. Anxiety and depression scores were generally lower in the TFCBT groups than in the SC groups.

**Conclusion:**

There is evidence for the effectiveness of TFCBT compared to SC in preventing chronic PTSD in patients with an initial ASD diagnosis. As this evidence originates from one research team replications are necessary to assess generalisability. The evidence about the effectiveness of TFCBT in traumatised populations without an ASD diagnosis is insufficient.

## Background

People exposed to stressful events may develop trauma-related psychiatric conditions. Clinicians, researchers and policymakers are increasingly interested in early interventions to prevent the development of chronic mental health problems such as post-traumatic stress disorder (PTSD) [1]. Psychological debriefing (PD) has perhaps been the most widespread among such early interventions. However, systematic reviews have failed to show any effectiveness of one-session PD when given to all exposed individuals [2-5]. Trauma-focused cognitive behaviour therapy (TFCBT) is the recommended early intervention for people with acute stress disorder (ASD) or acute PTSD in National Institute for Health and Clinical Excellence's guidelines [1]. The recommendation is based on a systematic review that included nine studies retrieved from a literature search in January 2004. There was limited and inconclusive evidence that TFCBT delivered between 1 and 6 months after trauma was more effective than being on a waiting list, or receiving alternative psychosocial interventions. Our objective was to provide an updated evaluation of the effectiveness of early TFCBT compared to other psychosocial interventions in preventing PTSD, anxiety and depression among adults with ASD or PTSD symptoms.

## Methods

This systematic review is based on two health technology assessments (HTAs) of early psychosocial interventions following traumatic events [6,7]. The HTAs were commissioned by the Norwegian Directorate for Health and Social Affairs to obtain an overview of all kinds of early psychosocial interventions following all types of traumatic events, and to use the evidence in the development of clinical guidelines. As the objective of this review is narrower than that of the HTAs, we have performed a new literature search and applied a refined set of study eligibility criteria.

We searched MEDLINE, Embase, PsycINFO, CINAHL, Cochrane Central Register of Controlled Trials (CENTRAL), ISI Web of Science and PILOTS, with each database being searched from inception to June or July 2007. We used subject headings and text words for PTSD symptoms and cognitive-behavioural therapy combined with Ovid's optimised search strategy for randomised trials developed and validated by the Health Information Research Unit at McMaster University [8]. The search was restricted to adult populations.

### Study selection

We included studies that met the following criteria:

- randomised controlled trial (RCT) published in peer-reviewed scientific journal

- a study population of adults with symptoms of acute stress disorder (ASD) or symptoms of post-traumatic stress disorder (PTSD)

- individual TFCBT initiated within three months post trauma

- a non-pharmacological comparison intervention

- outcomes measured as symptoms and/or diagnosis of PTSD (primary outcome), anxiety and/or depression (secondary outcomes) at follow up (minimum one month after treatment completion).

Individual TFCBT was defined as an intervention with at least four planned sessions, regardless of the number of sessions actually completed. At least one of the following techniques should be included in the intervention: exposure, systematic desensitization, stress inoculation training, cognitive processing therapy, cognitive therapy, assertiveness training, biofeedback, relaxation training. The minimum of four sessions was chosen to differentiate TFCBT from debriefing techniques, which may resemble TFCBT in some respects, but are given over one or two sessions only. We did not include studies that compared the effectiveness of TFCBT to conditions with no interventions, such as waiting list controls. There is, at least in Norway, a public expectation to offer some kind of mental health care to traumatised people, and many would perceive no intervention or delayed interventions as unethical. Therefore, our objective was to evaluate whether or not TFCBT was more effective than other interventions. We excluded studies in other languages than English, the Scandinavian languages, French and Italian. Independent pairs of reviewers selected studies for inclusion. A third reviewer was consulted to resolve any disagreement regarding inclusion decisions.

### Quality assessment

We assessed the methodological quality of included studies on the basis of randomisation, adequate concealment of randomisation, level of blinding, use of intention-to-treat (ITT) analysis and description of loss to follow up. Two reviewers assessed study quality independently using a checklist developed at the Norwegian Knowledge Centre for the Health Services, based on "User's Guides to the Medical Literature" [9].

### Data extraction

One reviewer extracted data from the studies into pre-designed data forms including study, patient and intervention characteristics, relevant outcome measures and study results. At least one other reviewer checked extracted data and any disagreement were resolved by discussion. We reported data from studies with multiple publications as a single study.

### Data synthesis

We used the Review Manager 4.2 software (Nordic Cochrane Centre, 2003) for meta-analysis where patients, interventions and outcomes were consistent enough across studies to justify pooling. Effect estimates were risk ratio (RR) and standardised mean difference (SMD) with 95% confidence intervals (CI) for dichotomous and continuous outcomes, respectively. We used SMD even when studies used the same assessment instrument for an outcome to accommodate the evaluations of clinical meaningfulness (see below). We used a fixed-effects model to calculate effect estimates when the I^2^-test for heterogeneity was less than 30%. Otherwise we used the random-effects model. We adhered to Bisson and coworkers' threshold criteria for clinically meaningful effect estimates when two active treatments are compared: SMD ≤ -0.5 or ≥ 0.5, and RR ≤ 0.80 or ≥ 1.25 [10]. Further, 95% confidence intervals for clinically meaningful effect estimates should not cross the thresholds.

## Results

**Figure 1:**
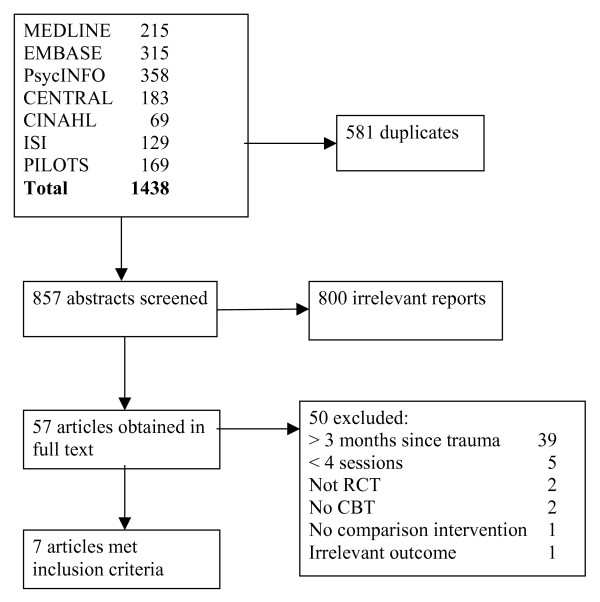
Flow chart of study inclusion process.

The searches identified 1438 studies, of which 857 remained after removal of duplicates (Figure 1). Fifty seven references appeared to meet our eligibility criteria and were obtained in full text. We included seven of these articles.

### Study characteristics

The seven articles originated from five different RCTs: in addition to one article for each trial, long-term follow-up outcomes for three of the RCTs [11-13] were reported in two articles [14,15] (Tables 1 and 2). The total number of randomised participants across studies was 257. There was a female majority (ranging from 50% to 100%) among the participants in all the trials and the average age ranged from 29 to 37 years. Participants had been exposed to motor vehicle or industrial accidents or assaults 2 – 46 days prior to study inclusion. Mean pre-treatment scores on the Impact of Event Scale (IES) indicated clinically significant levels of posttraumatic symptoms (IES Total > 19, Intrusion and Avoidance subscales > 9) [16].

**Table 1:** Characteristics of included studies

First author, publication year and country	Participants randomised/in post-treatment analyses, n	Age, years (s.d.)	Female %	Traumatic events and primary diagnosis	Time since trauma, days (s.d.)	TFCBT technique(s) and comparison intervention
Bryant 1998 Australia [11]	24/24	32 (12.6) 33 (11.4)	58	MVA Industrial accident ASD	10 (4.2) 10 (5.0) 7 (2.5)	Education, relaxation training, imaginal exposure, cognitive restructuring, in vivo exposure Supportive counselling
Bryant 1999 Australia [12]	56/41	33 (10.9) 33 (13.4) 37 (12.0)	50	MVA Nonsexual assault ASD	10 (3.3) 10 (4.1) 11 (3.4)	Education, imaginal exposure, cognitive restructuring, in vivo exposure TFCBT + anxiety management Supportive counselling
Bryant 2003a Australia [18]	24/24	29 (13.9) 33 (14.4)	67	MVA Nonsexual assault ASD	< 14	Education, relaxation training, imaginal exposure, cognitive restructuring, in vivo exposure Supportive counselling
Bryant 2003b Australia [14]	80/41	Follow-up of participants in Bryant 1998 and Bryant 1999				
Bryant 2005, Bryant 2006 Australia [13,15]	87/69/53	33 (12.5) 33 (7.7) 35 (13.3)	61	MVA Nonsexual assault ASD	16 (8.8) 14 (6.7) 14 (8.4)	Imaginal exposure, cognitive restructuring, in vivo exposure TFCBT + hypnosis Supportive counselling
Foa 2006 USA [17]	90/57/66	34 (11.1)	100	Sexual/nonsexual assault PTSD (not duration criterion)	21 (range: 2–46)	Education, relaxation training, imaginal exposure, cognitive restructuring, in vivo exposure Supportive counselling Assessments only

ASD, acute stress disorder (DSM-IV); PTSD, posttraumatic stress disorder (DSM-IV, not duration criterion); MVA, motor vehicle accident; TFCBT, trauma-focused cognitive-behavioural therapy; CIDI, Composite International Diagnostic Interview; IES, Impact of Event Scale; BDI, Beck's Depression Inventory; STAI, State/Trait Anxiety Inventory; CAPS-2, Clinician Administered PTSD Scale; BAI, Beck's Anxiety Inventory; PSS-I/-SR, PTSD Symptom Scale-Interview/-Self-Report; ITT, intention to treat; LOCF, last observation carried forward.

**Table 2:** Characteristics of included studies (continued)

First author, publication year and country	Treatment duration and provider competence	Outcome measures	Post-treatment follow-up	ITT analysis
Bryant 1998 Australia [11]	5 × 90 minutes Clinical psychologists	CIDI (PTSD diagnosis) IES (intrusion/avoidance) BDI (depression) STAI (state/trait anxiety)	6 months (post trauma)	Yes
Bryant 1999 Australia [12]	5 × 90 minutes Clinical psychologists	CAPS-2 (PTSD diagnosis) IES (intrusion/avoidance) BDI (depression) STAI (state/trait anxiety)	6 months	No (completers only)
Bryant 2003a Australia [18]	5 × 90 minutes Clinical psychologists	CAPS-2 (PTSD diagnosis) IES (intrusion/avoidance) BAI (anxiety) BDI (depression)	6 months	Yes
Bryant 2003b Australia [14]		CAPS (PTSD diagnosis)	4 years	Yes (LOCF)
Bryant 2005, Bryant 2006 Australia [13,15]	5 × 90 minutes Clinical psychologists	CAPS-2 (PTSD diagnosis/symptoms) IES (intrusion/avoidance) BAI (anxiety) BDI (depression)	6 months 3 years	Yes (LOCF)
Foa 2006 USA [17]	4 × 120 minutes Master's and doctoral level therapists	PSS-I/PSS-SR (PTSD diagnosis/severity) BAI (anxiety) BDI (depression)	2, 3, 6, 9 and 12 months	Completers only

ASD, acute stress disorder (DSM-IV); PTSD, posttraumatic stress disorder (DSM-IV, not duration criterion); MVA, motor vehicle accident; TFCBT, trauma-focused cognitive-behavioural therapy; CIDI, Composite International Diagnostic Interview; IES, Impact of Event Scale; BDI, Beck's Depression Inventory; STAI, State/Trait Anxiety Inventory; CAPS-2, Clinician Administered PTSD Scale; BAI, Beck's Anxiety Inventory; PSS-I/-SR, PTSD Symptom Scale-Interview/-Self-Report; ITT, intention to treat; LOCF, last observation carried forward.

All included studies compared TFCBT with supportive counselling (SC). The SC programs consisted of active listening and education about trauma and general problem-solving skills. Cognitive restructuring, exposure techniques and other forms of focusing on the individual's specific traumatic experience were avoided.

Two studies had an additional intervention arm: TFCBT + anxiety management [12] and TFCBT + hypnosis [13,15], respectively. In our view, anxiety management and hypnosis can be considered components of TFCBT. The additional intervention arms were therefore treated as TFCBT groups. We merged the pure TFCBT groups and TFCBT + anxiety management/hypnosis groups when we calculated the pooled relative risk for PTSD diagnosis (the only dichotomous outcome). In the analyses of continuous outcomes we pooled the means and standard deviations in the pure TFCBT arms and the TFCBT + additional component arms. Pooled means were calculated with the following formula: (mean_1 _*n_1_) + (mean_2 _*n_2_)/n_1 _+ n_2 _where n is number of participants, 1 is arm 1 and 2 is arm 2. Pooled standard deviations were calculated with the following formula:

$$s_{p}=\sqrt{\frac{(n_{1}-1)s_{1}^{2}+(n_{2}-1)s_{2}^{2}+\cdots +(n_{k}-1)s_{k}^{2}}{n_{1}+n_{2}+\cdots +n_{k}-k}}$$

where *s *is standard deviation, *p *is pooled, *n *is number of participants, 1 is arm 1, 2 is arm 2, *k *is arm k and *k *is number of arms.

One study used assessment only as a third study arm [17]. The assessment only condition was not entered into our meta-analysis, as we only included comparisons that were active treatments.

### Methodological quality

All the included RCTs had in common that the randomisation procedures were insufficiently described and that concealment of allocation was not addressed. Additional shortcomings of three of the RCTs [12,13,15,17] were a lack of intention to treat analysis and that dropouts were not accounted for.

### Clinical effectiveness

**Figure 2:**
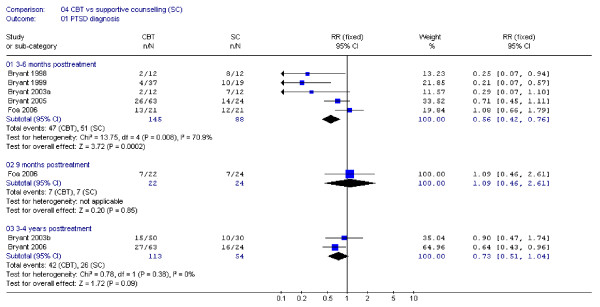
Meta-analysis of PTSD diagnosis at 3–6 months, 9 months and 3–4 years.

All five RCTs reported PTSD diagnosis at 3–6 months post treatment. Thirty two percent (47 of 145 participants) in the TFCBT groups had a PTSD diagnosis versus 58% (51/88) in the SC groups (Figure 2). The effect estimate was statistically significant, but the study results were heterogeneous. We performed post hoc subgroup analyses to explore the heterogeneity. An analysis excluding Foa and coworkers' study [17] did not remove heterogeneity (I^2 ^= 61%), but when we also excluded Bryant and coworkers' 2005 study [13] homogeneity was achieved (I^2 ^= 0%). Combining the Foa and the Bryant 2005 studies only resulted in I^2 ^= 35%.

The PTSD rates were 32% (7/22) in the TFCBT group versus 29% (7/24) in SC group at 9 months [17], and 37% (42/113) versus 48% (26/54) after 3–4 years [14,15]. The 9-month and 3–4-year effect estimates were not statistically significant.

Bryant and coworkers' four studies reported mean scores on IES subscales Intrusion and Avoidance at 6 months follow-up. The TFCBT groups' mean Intrusion and Avoidance scores were significantly lower than those of the SC groups. The SMDs were statistically significant, but there was mild heterogeneity between Avoidance estimates.

Foa and coworkers [17] reported interviewer-rated and self-rated PTSD severity at 3 and 9 months post-treatment. Group differences varied in directions and were not statistically significant. All included studies reported symptom levels for anxiety and depression at 3–6 months follow-up: there was tendency towards lower symptom levels for TFCBT than for SC. The tendency was confirmed for depression symptoms by one study at 3 years follow-up [14].

### Clinical meaningfulness

None of the effect estimates met the criteria we had selected for clinical meaningfulness (Table 3). There was limited evidence for the superior effectiveness of TFCBT compared to SC on PTSD when assessed 3–6 and 36–48 months post treatment, on IES subscales Intrusion and Avoidance and on anxiety when assessed 3–6 months post treatment and depression assessed 36 months post treatment. There was inconclusive evidence for the superiority of TFCBT compared to SC on self-rated PTSD severity, on anxiety 9 months post treatment and depression 3–6 and 9 months post treatment. The evidence was also inconclusive for the superiority of SC compared to TFCBT on PTSD diagnosis and interviewer-rated PTSD severity 9 months post treatment.

**Table 3:** Summary of meta-analyses

Outcome	Months post-treatment	Studies	n	Effect estimate (95% CI)	Heterogeneity (I^2^)
PTSD diagnosis	3–6	[11-13,17,18]	233	RR 0.49 (0.25 to 0.94)	71%
	9	[17]	46	RR 1.09 (0.46 to 2.61)	---
	36–48	[14,15]	169	RR 0.73 (0.51 to 1.04)	0%
Impact of Event Scale: Intrusion	6				
		[11-13,18]	176	SMD -0.63 (-0.95 to -0.30)	0%
Avoidance		[11-13,18]	176	SMD -0.87 (-1.47 to -0.27)	65%
Interviewer-rated PTSD severity	3	[17]	42	SMD -0.22 (-0.83 to 0.38)	---
	9	[17]	46	SMD 0.11 (-0.47 to 0.69)	---
Self-rated PTSD severity	3	[17]	38	SMD -0.35 (-0.99 to 0.30)	---
	9	[17]	46	SMD -0.18 (-0.76 to 0.40)	---
Anxiety	3–6	[11-13,17,18]	214	SMD -0.56 (-0.85 to -0.27)	0%
	9	[17]	45	SMD -0.42 (-1.01 to 0.17)	---
Depression	3–6	[11-13,17,18]	217	SMD -0.34 (-0.62 to -0.05)	20%
	9	[17]	45	SMD -0.08 (-0.66 to 0.50)	---
	36	[15]	53	SMD -0.97 (-1.59 to -0.35)	---

PTSD, post-traumatic stress syndrome; RR, risk ratio; SMD, standardised mean difference; TFCBT, trauma-focused cognitive-behavioural therapy; SC, supportive counselling

However, clinical meaningfulness was achieved for PTSD at 3–6 months in a post hoc subgroup analysis of the four Bryant studies only (RR 0.36; 95% CI 0.17 – 0.78).

## Discussion

We identified and included seven articles reporting results from five randomised controlled trials comparing the effects of TFCBT with supportive counselling (SC). The evidence was not conclusive, but there was a tendency towards TFCBT being more effective than SC in preventing PTSD, and reducing PTSD, anxiety and depression symptoms. The tendency for TFCBT to be more effective than SC supports the findings in the systematic review that the National Institute for Health and Clinical Excellence's guidelines are based on [1].

Four of the included studies [11-15,18] had been carried out with very similar research protocols in Australia by Richard Bryant and his research team, while Edna Foa and her team in the USA were responsible for the fifth study [17]. The Australian and the US populations differed in several ways. The Australian population was mixed with regard to sex and type of trauma exposure, and time since trauma was less than 14 days at first assessment. The US population was female assault victims assessed 2–46 days after trauma. More importantly, a DSM-IV acute stress disorder (ASD) diagnosis was an inclusion criterion in the Australian studies, while a DSM-IV PTSD diagnosis disregarding the duration criterion was sufficient in the US study. As the ASD diagnosis requires the presence of at least three dissociative symptoms [19], the Australian study participants were more severely affected by the traumatic events, and it seems appropriate to assume that this important difference in study populations influenced treatment effectiveness on chronic PTSD.

The meta-analysis of PTSD at 3–6 months showed a heterogeneity that might reflect the population differences: there was a lower PTSD prevalence in the Australian TFCBT groups than in the SC groups, while there was no difference between the US groups. When we removed the Foa study from the meta-analysis we obtained a clinically meaningful effect estimate and evidence for TFCBT's superiority over SC in preventing chronic PTSD.

However, it was somewhat surprising that results still were heterogeneous in the subgroup analysis of the Bryant studies only. It seems like the largest and most recent Bryant study [13] accounted for the heterogeneity, as homogeneity was achieved on removal of that study from the subgroup analysis. The authors of the original paper point out the high attrition rate in the TFCBT groups as a possible explanation for the lack of a treatment effect in the Bryant 2005 study. Apparently, attrition was more evenly distributed in the other Bryant studies.

## Conclusion

TFCBT had limited effectiveness in preventing chronic PTSD in a clinically heterogeneous population. However, the evidence for the effectiveness of TFCBT in individuals with ASD seems clinically meaningful enough to have implications for practice. It supports an approach where mental health care facilities screen recently traumatised patients for ASD and consider offering TFCBT to those with a diagnosis. Replications are necessary to evaluate the effectiveness of TFCBT outside Australia, where all existing trials with ASD patients have been conducted.

## Competing interests

The authors declare that they have no competing interests.

## Authors' contributions

HK coordinated the review, drafted and revised all versions of the manuscript, screened and selected articles, assessed study quality, and extracted and entered data on trial results into RevMan. DW, ØE, LW and KJ contributed equally to conceiving and designing the review, drafting and editing the protocol, interpreting results and revising manuscript drafts. IK developed the search strategy, undertook searches of electronic databases, contributed to study selection and revised manuscript drafts. AS contributed to study selection, study quality assessments and interpreting results, and revised manuscript drafts. All authors have read and approved this manuscript.

## Pre-publication history

The pre-publication history for this paper can be accessed here:


